# Obituary, Etc.

**Published:** 1866-02

**Authors:** 


*Obituary.—Death of Prof. W. B. Herrick.*

We extract from the columns of the *Chicago Tribune* the following notice of the death of Prof. William B. Herrick, M. D., who, for a number of years subsequent to the commencement of the second volume of this JOURNAL in 1846, was one of the prominent members of its editorial staff:

On the last day of December, 1865, in the Insane Asylum at Augusta, Me., died Dr. William B. Herrick, formerly of this city. The intelligence of this painful termination of an useful and honored life will be received with sadness by many of our older citizens, to whom deceased was well known as the possessor of rare personal and professional qualities, demonstrated during the years of surgical career in Chicago. Dr. Herrick, at the time of his death, was about fifty years of age. He was a native of Lewiston Falls, Me. Receiving a liberal education, he came to Illinois a quarter of a century ago, and for several years accumulated a practice and reputation in the interior of the State, whence he was called about the year 1845 to fill a chair in Rush Medical College in this city. Few who have been associated with him, in the office of medical instructor, ever exceeded him in the influence he had upon his students by the zealous and high-toned ardor that characterized his quest of science, and his own enthusiasm he was skilled in communicating to those who in his guidance pursued the paths of learning. At the outbreak of the Mexican war, Dr. Herrick temporary vacated his chair, and entered the service as surgeon of an Illinois regiment. It is volumes to say of the reputation that he received in the field, when it is stated that at the expiration of the term of his voluntary enlistment, the close of the war, the Medical Bureau of the Government with great regret received his declination of the rare compliment of an appointment to the medical staff of the regular army. But Dr. Herrick's heart was with his college, and he came back with his qualities ripened and strengthened to fill an even more distinguished rank in his coveted place among his fellows. Like too many of those who with him escaped the actual presence of death in the terrible campaigns and climate of Mexico, the destroyer followed him home to prey leisurely but surely upon his life. Struggling against the disease with all the appliances of skill, his chronic neuralgia became paralysis, terminating in a softening of the brain, reducing the strong framed and acute minded man to the bodily and mental wreck upon which the office of death seemed less kind because delayed through seven long years of darkness. In the loss of Dr. Herrick, dating from the beginning of the painful eclipse of his faculties, the cause of science in the West lost a noble and zealous votary, and our community a valued and generous citizen.

---

Many of our readers will be pleased to hear that Dr. R. M. Lackey has been appointed Demonstrator of Anatomy in Rush Medical College, in place of Prof. E. Powell resigned. Dr. Lackey has filled with no ordinary distinction for more than four years various positions in the medical service of the army during the late war. He returns with rich experience in the practice of surgery and medicine, not only in the field but in the hospital.

---

The first semi-annual meeting of the Central States Association of Dental Surgeons will be held in the city of Nashville, Tenn., commencing on Tuesday, April 10th, at 10 o'clock, A. M.

---

VACCINE VIRUS.—Physicians in the country can obtain healthy and reliable virus, by forwarding $1 to R. M. LACKEY, M. D., P. O. Box 2175, Chicago.

